# A Catalogue of Quacks

**Published:** 1905-02-11

**Authors:** 


### A Catalogue of Quacks.

The columns of *Truth* excite varied sentiments in the minds of politicians, but Whig and Tory all agree in offering tribute to the Editor of our contemporary on his unceasing and energetic crusade against the numerous tribe of quacks and humbugs who prey on an ignorant and gullible public. In a recent issue he renders further service in this direction by publishing a list of various individuals, firms, and organisations upon whom, in the course of 1904, he has had to make unfavourable comment. The length of that list is a saddening index of the ease with which the British public can be exploited by the unscrupulous. To hope that such an exposure will be entirely successful would be contrary to the teachings of experience. Yet it surely cannot completely fail, and the careful investigations which must have preceded it and the courage necessary for its publication constitute a notable service which well deserves recognition. Of special interest to the readers of our columns is the section of the catalogue devoted to medical quacks, and it is not pleasant to know from so well-informed a source that the number of these during 1904 has shown "an alarming increase." A possible explanation is found in the fact that in the United States these gentry are now prevented from using the national post-office for the purpose of their business, and that they are therefore availing themselves to an increasing extent of the unrestricted facilities which the British Government places at their disposal. A contributing cause is doubtless found in the readiness with which many popular journals—and some of the so-called religious papers occupy in this connection a bad eminence—accept and distribute advertisements without adequate supervision of the statements these contain. It is, indeed, sufficient to glance over *Truth's* black list and to recall advertisement columns that come almost daily under observation to see how repeatedly proprietors of these prints undertake some degree of moral responsibility for the success of undertakings which have been shown to be grossly fraudulent. The plea of ignorance in this respect, if at any time an excuse, is an excuse no longer. The record collected by our contemporary is a very comprehensive one, and it is phrased in terms which none can affect to misunderstand. Turning to the list itself we find many familiar names both of individuals and "institutes," and we receive information of their origin and nature which is of the first importance. It is not very infrequent for medical practitioners to be appealed to by members of the public in regard to several of the names here catalogued, and in the absence of precise information there is not unnaturally some hesitation in pronouncing a specific condemnation, more especially as many foolish persons attribute such a verdict to professional jealousy. Hence we will venture to advise our readers to file the black list of quacks which *Truth* provides. They will thus be provided with information not only of interest to themselves, but useful in protecting their patients against the wiles of a series of nefarious humbugs. They will learn that a much-advertised "Institute for the Deaf" has been described by a British judge as "a disgraceful institution carried on for unworthy objects by discreditable means"; that a company claiming to sell special foods for diabetics prepared at their own mills in America, supplies samples obtained from a London firm; and that it actually pays some person to advertise a "gymnasium for the eye," claiming to cure all forms of eye disease at a cost to the dupe of £2 5s. Surely in this the limit of human credulity must have been reached.

Another useful side-light on the method of the quack fraternity is a statement of the frequency with which one and the same individual advertises his wares under different loud-sounding names. An artificial ear-drum, a superfluous hair destroyer, a female bust improver, an "anti-fat" tablet, and a notorious drink cure, though each modestly presented to the public as owning a separate origin, may all, it seems, be traced to one and the same ingenious mind.

We again commend this catalogue of quacks to the attention of our readers, and we will suggest that they may render a public service by offering an emphatic protest should the name and pretensions of any one of the number appear in the advertisement columns of any journal which they keep on their list of periodicals.

